# Left Atrial Mechanics and Remodeling in Paroxysmal Atrial Fibrillation: Introducing the EASE Score for Pre-Ablation Risk Prediction

**DOI:** 10.3390/medsci13030131

**Published:** 2025-08-14

**Authors:** Fulvio Cacciapuoti, Ilaria Caso, Rossella Gottilla, Fabio Minicucci, Mario Volpicelli, Pio Caso

**Affiliations:** 1 Division of Cardiology, “A. Cardarelli” Hospital, 80131 Naples, Italy; 2 Department of Cardiology, “V. Monaldi” Hospital, 80131 Naples, Italy; 3 Department of Cardiology, “San Leonardo” Hospital, 80131 Naples, Italy; 4 Division of Arrhythmology, “Santa Maria della Pietà” Hospital, 80131 Naples, Italy

**Keywords:** paroxysmal atrial fibrillation, left atrial remodeling, echocardiographic risk model, atrial strain imaging, electromechanical delay, catheter ablation outcome

## Abstract

**Background:** Paroxysmal atrial fibrillation (PAF) is a common arrhythmia often treated with catheter ablation, particularly pulmonary vein isolation (PVI). However, recurrence remains frequent and is often linked to unrecognized structural and functional remodeling of the left atrium. **Methods:** We introduce the Echocardiographic Atrial Strain and conduction Evaluation (EASE) score as a theoretical, noninvasive model to stratify recurrence risk in patients undergoing catheter ablation for PAF. The score is based on the hypothesis that integrated echocardiographic parameters can reflect the extent of atrial remodeling relevant to ablation outcomes. **Results:** The EASE score combines six echocardiographic metrics—left atrial reservoir strain (LASr), atrial conduction time (PA-TDI), left atrial volume index (LAVI), stiffness index (E/e′/LASr), E/e′ ratio, and contractile strain (LASct)—each representing structural, electrical, or mechanical remodeling. The total score ranges from 0 to 12, stratifying patients into low, intermediate, and high-risk categories for arrhythmia recurrence. Preliminary retrospective data suggest a significant association between higher EASE scores and increased recurrence rates following ablation. **Conclusions:** The EASE score offers a biologically plausible, multidimensional framework for noninvasive risk prediction in PAF ablation. Prospective studies are warranted to validate its clinical utility and refine its structure.

## 1. Introduction

Paroxysmal atrial fibrillation (PAF) is a common cardiac arrhythmia characterized by episodes of irregular atrial activity that begin abruptly and terminate spontaneously, typically within 48 h and always within seven days [1]. Despite its transient presentation, PAF is associated with a substantial clinical burden, including elevated risks of stroke, heart failure, and progression to persistent or permanent atrial fibrillation [2]. It also significantly impairs quality of life and increases healthcare utilization.

Catheter ablation, particularly pulmonary vein isolation (PVI), has become a cornerstone treatment for symptomatic, drug-refractory PAF [3]. By targeting the pulmonary vein triggers that initiate atrial fibrillation episodes, ablation offers the potential for rhythm control and symptom relief [4]. However, recurrence after ablation remains a significant challenge, affecting a considerable proportion of patients [5]. Variability in outcomes is often attributed to the underlying degree of atrial remodeling, which may be underestimated or undetected using standard clinical assessments [6].

In this context, echocardiography, particularly advanced modalities such as speckle-tracking and tissue Doppler imaging, has emerged as a valuable tool for characterizing atrial structure and function [7]. Echocardiographic parameters can reveal early signs of atrial myopathy, including fibrosis, dilation, and contractile dysfunction, all of which influence the likelihood of ablation success [8].

This manuscript presents a testable clinical hypothesis regarding a novel echocardiographic scoring system, the Echocardiographic Atrial Strain and conduction Evaluation (EASE) score, that may improve risk stratification in AF ablation candidates. By integrating six key parameters reflecting electrical, mechanical, and structural remodeling, the EASE score could provide a practical, noninvasive framework to guide clinical decision-making and personalize patient care.

## 2. Pathophysiological Background

PAF arises from a complex interaction between focal electrical triggers and an evolving atrial substrate [9]. In the majority of cases, the initial episodes of PAF are driven by ectopic activity originating in the pulmonary veins [10]. However, as the condition progresses, structural and functional remodeling of the atrial myocardium begins to play a more prominent role in arrhythmia maintenance and recurrence [11].

The atrial remodeling process is multifaceted. Structurally, it involves progressive enlargement of the left atrium, increased atrial wall thickness, and interstitial fibrosis [12]. These changes reduce atrial compliance, impair mechanical function, and disrupt normal conduction pathways [13]. Functionally, remodeling leads to altered atrial contractility and diminished reservoir and conduit phases of left atrial function. Electrically, there is a slowing of conduction, increased dispersion of refractoriness, and the potential for reentrant circuits, all of which enhance the substrate for sustained atrial fibrillation [14].

Inflammation, oxidative stress, neurohormonal activation, and elevated atrial pressures all contribute to the remodeling cascade, especially in obesity and diabetes [15]. Moreover, conditions such as hypertension, heart failure, and sleep apnea can accelerate these changes and further destabilize atrial electrophysiology [16].

Understanding the underlying pathophysiology of atrial remodeling and its mechanical effects is essential for optimizing the timing and strategy of catheter ablation. While focal pulmonary vein isolation addresses the initiating triggers, the degree of atrial remodeling ultimately influences long-term success [17].

Importantly, the accumulation of epicardial adipose tissue (EAT), particularly in the left atrial region (LA-EAT), has emerged as a potent contributor to atrial remodeling. LA-EAT influences atrial function through paracrine inflammatory signaling, mechanical compression, and promotion of fibrosis. Its presence is associated with reduced atrial strain, elevated stiffness, and regional conduction heterogeneity [18]. While EAT volume is not directly included in the EASE score, its effects are indirectly captured through the strain and stiffness parameters it includes.

Hence, a reliable, noninvasive method to assess the structural and functional integrity of the left atrium, such as the EASE score, could provide critical insights into patient selection and expected procedural outcomes.

## 3. Components of the EASE Score

**Figure 1:**
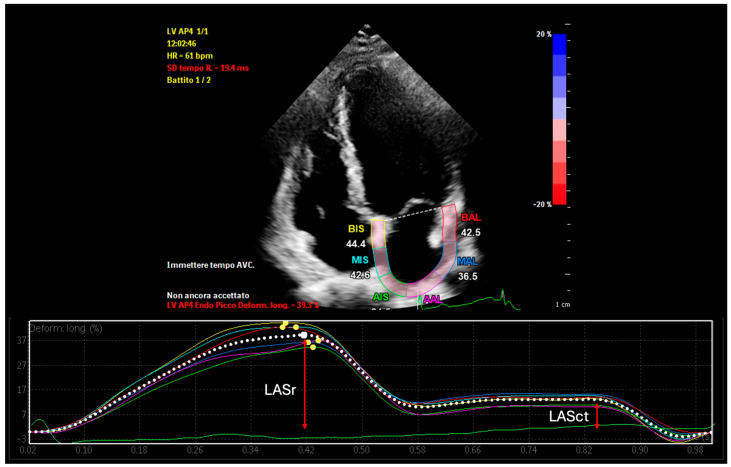
Left Atrial Reservoir Strain (LASr) and Left Atrial Contractile Strain (LASct) measurement in a healthy subject.

Left Atrial Reservoir Strain (LASr): LASr is a measure of atrial deformation during ventricular systole, reflecting the reservoir function of the left atrium [19]. It is assessed using 2D speckle-tracking echocardiography and reported as a percentage (Figure 1). A normal LASr value is typically greater than 23%, indicating good atrial compliance and minimal fibrosis. Values between 15% and 23% are considered borderline, while values below 15% suggest advanced atrial remodeling [20]. Reduced LASr has been associated with atrial fibrosis, decreased left atrial compliance, and a higher risk of recurrence after catheter ablation [21].

**Figure 2:**
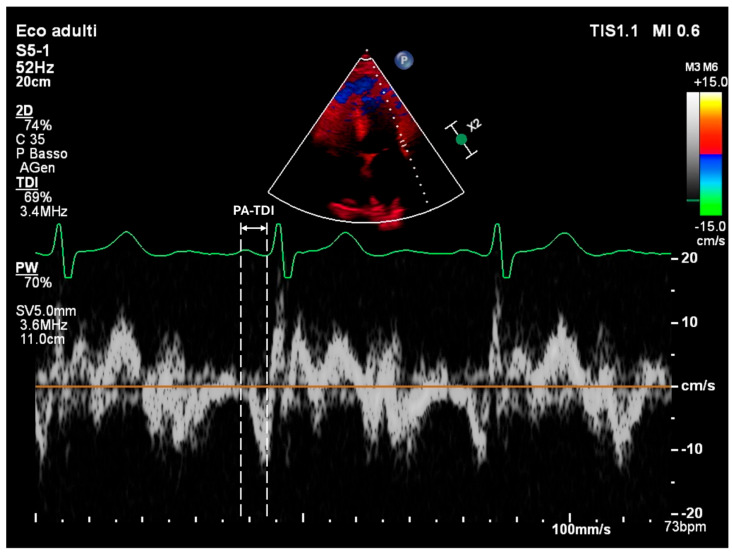
PA-TDI measurement in a healthy subject. Assessing left atrial mechanical function.

Atrial Conduction Time (PA-TDI): PA-TDI is calculated as the time interval from the onset of the P wave on a surface ECG to the onset of the A′ wave measured by tissue Doppler imaging at the lateral mitral annulus [22] (Figure 2). This parameter reflects atrial electromechanical delay and serves as an indicator of electrical remodeling. Normal values are usually below 120 msec, while intervals above 150 msec suggest significant conduction slowing, often due to fibrotic tissue or dilated atrial architecture [23]. Prolonged PA-TDI is independently associated with arrhythmia persistence and ablation failure [24].

**Figure 3:**
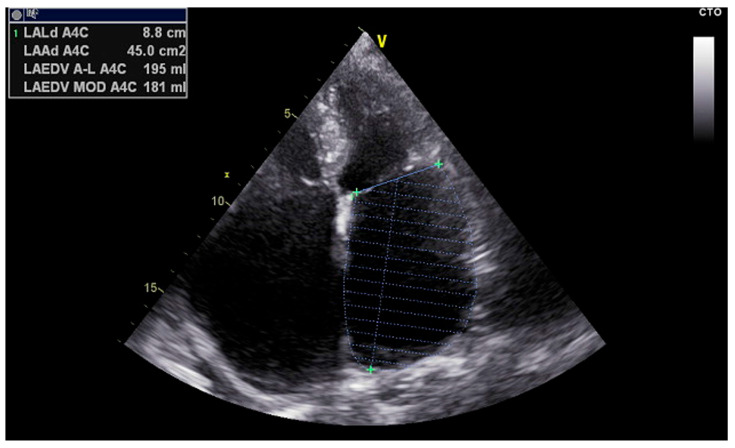
Left atrial volume measurement using Simpson’s biplane method.

Left Atrial Volume Index (LAVI): LAVI quantifies the size of the left atrium indexed to body surface area and is measured using the biplane method of disks (modified Simpson’s rule) from apical four- and two-chamber views [25] (Figure 3). Normal LAVI is below 34 mL/m^2^, with values between 34 and 48 mL/m^2^ representing mild to moderate enlargement, and values above 48 mL/m^2^ indicating severe dilation. Increased LAVI reflects chronic pressure or volume overload, often linked to diastolic dysfunction or longstanding atrial fibrillation [26]. It is a robust predictor of adverse cardiovascular events and procedural failure in AF ablation.

Left Atrial Stiffness Index (E/e′/LASr): This index combines two critical metrics: E/e′, which reflects left ventricular filling pressure, and LASr, which reflects atrial compliance [27]. The stiffness index is calculated by dividing E/e′ by LASr. A value below or equal to 0.5 indicates normal stiffness, whereas a value above 0.5 denotes increased left atrial stiffness. Elevated stiffness is indicative of impaired reservoir function and advanced remodeling, and has been shown to correlate with post-ablation recurrence [28].

E/e′ Ratio: The E/e′ ratio is a widely used echocardiographic parameter for estimating left ventricular diastolic pressures [29]. It is derived from early mitral inflow velocity (E wave) and early diastolic mitral annular velocity (e′) obtained via pulsed-wave and tissue Doppler imaging, respectively. A ratio above 14 suggests elevated left atrial pressure and diastolic dysfunction [30]. These changes contribute to left atrial strain and enlargement, providing an arrhythmogenic substrate for AF maintenance [31].

Left Atrial Contractile Strain (LASct): LASct represents the active contraction function of the left atrium and is evaluated using speckle-tracking echocardiography during the late diastolic phase [32] (Figure 1). It is measurable only in patients who are in sinus rhythm at the time of examination. Normal values are typically above 6%, whereas values below this threshold suggest poor contractile function and advanced atrial disease [33]. Impaired LASct has been linked to a greater likelihood of arrhythmia recurrence and reflects the diminished booster pump contribution of the atrium to left ventricular filling [34].

**Figure 4:**
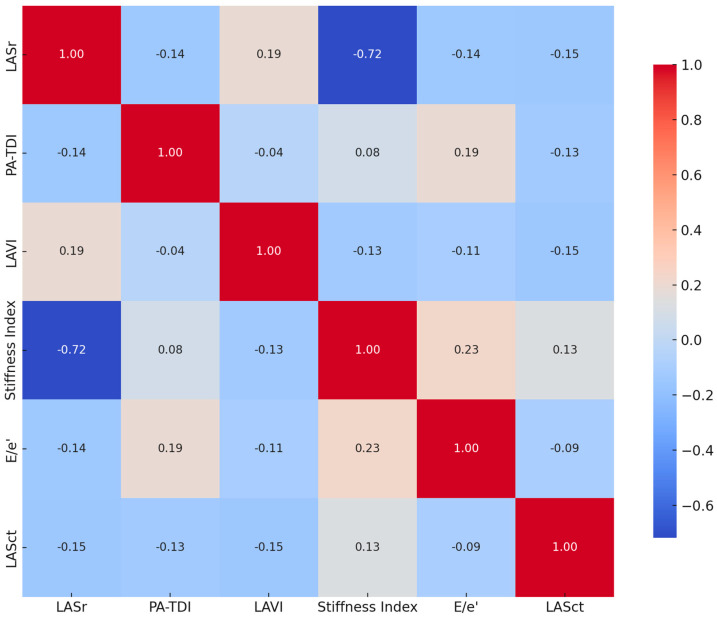
Pearson correlation heatmap of EASE score parameters. LASr demonstrates a strong negative correlation with the stiffness index (r = −0.72), while other parameters such as PA-TDI, LAVI, and LASct show relatively weak correlations, supporting the additive and non-redundant value of each component.

These six parameters, when evaluated collectively, provide a multidimensional assessment of left atrial structure, function, and electromechanical integrity. Their integration into the EASE score enables precise risk stratification (Figure 4) and supports a tailored approach to the management of patients undergoing AF ablation (Table 1).

Based on the distribution of EASE scores in our study cohort and their association with arrhythmia recurrence, we classified patients into three risk groups: low (0–3), intermediate (4–8), and high (9–12). This stratification was determined empirically by identifying score intervals associated with progressively higher recurrence rates during follow-up. Patients in the low-risk group exhibited significantly lower recurrence rates (<10%), while those in the high-risk group demonstrated recurrence rates > 60%. The intermediate group represented a transitional zone with moderate risk. This tripartite model facilitates clinical decision-making by providing a practical, biologically grounded framework to estimate recurrence probability and tailor post-ablation management.

## 4. Echocardiographic–Electrophysiological Correlation

The EASE score’s predictive strength lies in its ability to noninvasively capture pathophysiological remodeling processes that correspond to the electrical behavior of the left atrium observed during invasive electrophysiological (EP) mapping. Each component of the score correlates with specific features of the arrhythmogenic substrate, offering a mechanistic bridge between imaging and intervention (Table 2).

**Figure 5:**
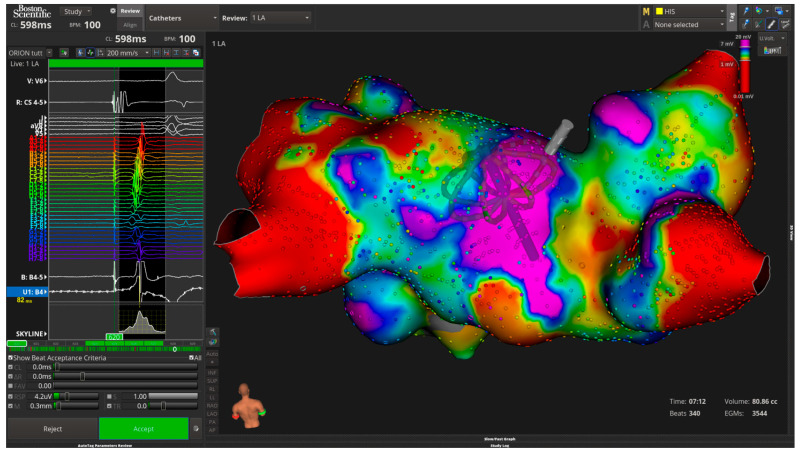
Electroanatomical voltage map of the left atrium showing extensive low-voltage areas (purple), indicative of atrial fibrosis and remodeling consistent with high EASE score risk.

Left Atrial Reservoir Strain (LASr) is perhaps the most sensitive echocardiographic indicator of myocardial fibrosis and compliance [35]. Low LASr values (<15%) consistently correspond to regions of low-voltage myocardium (<0.5 mV) on bipolar voltage mapping, representing fibrotic or non-viable atrial tissue [36] (Figure 5). These regions often exhibit sparse or absent Complex Fractionated Atrial Electrogram (CFAE) activity, reduced local capture during pacing, and decreased ablation responsiveness, particularly when confined to the posterior and inferior walls [37].

Atrial Conduction Time (PA-TDI) reflects electromechanical delay and is a noninvasive surrogate for slowed intra-atrial conduction. Patients with prolonged PA-TDI (>150 ms) often display broader total activation time during sinus rhythm or pacing, interatrial dyssynchrony, and delayed left atrial appendage activation [38]. These features align with conduction block or zig-zag conduction patterns on EP mapping, indicating an arrhythmogenic milieu that favors reentry [39].

Left Atrial Volume Index (LAVI) represents cumulative structural remodeling. Atrial dilation, particularly when indexed LAVI exceeds 48 mL/m^2^, is associated with greater spatial dispersion of CFAEs and more complex propagation patterns during AF [40]. Mapping in these patients frequently reveals multiple breakthrough sites, longer electrogram duration, and a wider distribution of rotors or wavelets.

Left Atrial Stiffness Index (E/e′/LASr) integrates diastolic burden and reservoir dysfunction. Elevated stiffness index values (>0.5) correlate with increased atrial afterload, loss of compliance, and the development of conduction heterogeneity [41]. These areas often overlap with low-voltage zones and regions exhibiting fragmented or double potentials, particularly in the posterior wall.

E/e′ ratio reflects elevated left atrial pressures and indirectly contributes to remodeling via hemodynamic stress. High E/e′ (>14) has been associated with greater AF vulnerability and difficulty maintaining sinus rhythm post-ablation. EP correlates include prolonged local electrogram duration and lower voltage density [42].

Left Atrial Contractile Strain (LASct), which represents active atrial systole, shows a strong association with late atrial potentials and mechanical booster function. LASct < 6% is predictive of atrial standstill or severely blunted contraction on EP recordings. These patients often demonstrate diffuse electrical quiescence in sinus rhythm and may fail to maintain mechanical recovery post-ablation [43].

Collectively, these echocardiographic parameters provide a multidimensional portrait of the atrial substrate. Their correlation with invasive findings validates the EASE score as a reliable predictor of electrophysiological remodeling. By identifying patients with extensive conduction delay, fibrosis, and mechanical impairment, the score offers practical guidance for tailoring ablation strategy, such as determining the need for posterior wall modification, targeting non-pulmonary triggers, or deferring ablation in favor of upstream therapy.

Reduced LASr aligns with zones of low-voltage and diminished CFAE density, indicating a fibrotic and non-conductive atrial substrate [44].

Prolonged PA-TDI corresponds with delayed conduction and increased atrial activation time [45].

While elevated LAVI is linked to expanded CFAE distribution and increased procedural complexity [46], a high stiffness index correlates with prolonged AF duration and reduced success of PVI [47].

Finally, E/e′ elevation reflects elevated filling pressure, which contributes to structural strain and diastolic stress, and an impaired LASct is associated with attenuated atrial late potentials and reduced sinus rhythm maintenance [48].

The incorporation of these findings into a composite model enhances predictive accuracy by capturing the multifactorial nature of atrial remodeling, including metabolic-inflammatory influences such as LA-EAT [49]. This indirect sensitivity to adipose-induced pathology extends the score’s relevance beyond purely structural metrics.

## 5. Discussion

The EASE score represents a significant advancement in the noninvasive assessment of atrial substrate for patients undergoing catheter ablation for paroxysmal atrial fibrillation. Traditional risk stratification methods often rely on clinical variables or singular echocardiographic measures, such as left atrial volume [50]. However, atrial fibrillation is a multifactorial disease involving structural, electrical, and mechanical remodeling of the atrium [51]. The EASE score could help address this complexity by integrating six distinct echocardiographic parameters, each targeting a different dimension of atrial health.

One of the key strengths of the EASE score lies in its comprehensive scope. Parameters such as LASr and LASct evaluate the mechanical function of the atrium during both passive filling and active contraction phases [52]. PA-TDI serves as a surrogate for conduction velocity and interatrial synchrony [53], while LAVI captures long-term structural adaptation to volume and pressure overload [54]. The inclusion of the LA stiffness index and the E/e′ ratio further contextualizes atrial function in the setting of diastolic load and compliance, which are highly relevant to atrial remodeling and fibrosis [55].

Importantly, all six components of the EASE score can be measured using standard transthoracic echocardiography with the addition of speckle-tracking and tissue Doppler imaging, modalities that are widely available in modern echocardiographic laboratories. This ensures broad applicability and reproducibility without reliance on invasive testing or advanced imaging techniques such as cardiac MRI.

From a clinical perspective, the EASE score has the potential to help clinicians in identifying patients who are most likely to benefit from ablation, informing discussions about procedural risks and expectations, and optimizing patient selection for early intervention versus more conservative management. High-risk patients, as identified by elevated EASE scores, may warrant more aggressive substrate mapping, closer post-procedural monitoring, or even consideration of alternative strategies.

Moreover, the EASE score provides a quantitative framework that may harmonize research efforts and improve standardization in clinical trials assessing outcomes of atrial fibrillation ablation. Its potential role extends beyond baseline assessment, offering a basis for longitudinal tracking of atrial remodeling in response to therapy or lifestyle modification.

By reflecting the true multidimensional nature of atrial health, the EASE score could enhance clinical decision-making and has the potential to improve procedural outcomes and patient care.

## 6. Preliminary Data and Validation Outlook

Although the EASE score has not yet undergone large-scale prospective validation, preliminary observational data from our institution suggest promising clinical utility. In a retrospective analysis of 128 patients who underwent first-time pulmonary vein isolation for paroxysmal atrial fibrillation between January 2020 and December 2023 (Table 3), higher composite EASE scores were significantly associated with increased arrhythmia recurrence at 12 months.

**Figure 6:**
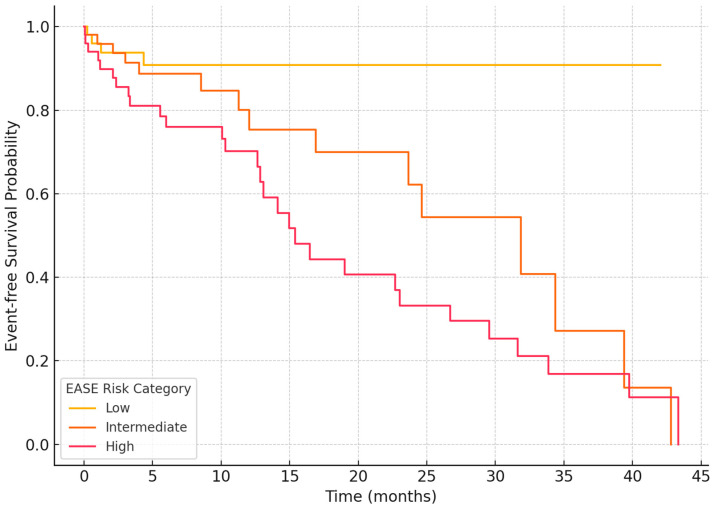
Kaplan–Meier curve showing arrhythmia-free survival stratified by EASE score categories (low: 0–3, intermediate: 4–7, high: 8–12).

The median follow-up duration was 14 months (IQR: 11–18 months). Arrhythmia recurrence was defined as any documented episode of atrial fibrillation, atrial flutter, or atrial tachycardia lasting longer than 30 s, occurring after a 3-month post-ablation blanking period. Notably, patients classified as high-risk (score ≥ 9) experienced more than twice the recurrence rate compared to those in the low-risk group (score ≤ 3). These preliminary findings support the EASE score’s potential as a discriminative tool for stratifying procedural outcomes (Figure 6). Nonetheless, these results are exploratory and based on a single-center retrospective cohort. Prospective, multicenter validation studies are warranted to confirm these associations and to refine the score’s thresholds and weighting.

## 7. Limitations and Future Directions

Although the EASE score could represent an innovative and practical tool for noninvasive risk stratification in patients undergoing catheter ablation for paroxysmal atrial fibrillation, its current formulation has some inherent limitations. At present, the score remains a conceptual model, lacking prospective validation through clinical trials. Without such validation, its predictive accuracy and generalizability remain hypothetical. Additionally, the use of speckle-tracking echocardiography and tissue Doppler imaging introduces a degree of operator dependency and variability in measurements, potentially affecting reproducibility. This is particularly relevant in clinical settings where echocardiographic expertise and image quality may differ significantly. As a conceptual hypothesis, the EASE score warrants prospective validation before routine clinical application.

Another important consideration is that some of the parameters included in the score, such as LASct, are rhythm-dependent and can only be reliably measured when the patient is in sinus rhythm [56]. This limits the applicability of the score in patients presenting with atrial fibrillation, who may be among those in greatest need of accurate risk stratification. In addition, we acknowledge that parameters such as LASr, LASct, and PA-TDI are subject to inter- and intra-observer variability. However, when performed by trained operators following standardized protocols, these measures have demonstrated acceptable reproducibility. Prior studies report intra-observer intraclass correlation coefficients > 0.80 for LASr, and >0.85 for PA-TDI when measured under appropriate image quality and acquisition settings. Therefore, structured training and adherence to guidelines [57] are essential for consistent application of the EASE score in clinical practice.

Furthermore, while the score reflects the influence of EAT through its impact on atrial strain and stiffness, it does not include a direct measure of EAT or atrial fibrosis, both of which are increasingly recognized as critical contributors to arrhythmogenic remodeling. Incorporating imaging modalities such as cardiac MRI or CT could help address this gap [58,59].

Moreover, the current version of the EASE score assigns equal weight to each of its six parameters, an approach based more on theoretical rationale than statistical optimization. Future studies should aim to refine the scoring system using outcome-driven data to establish more accurate weighting and threshold definitions.

Looking forward, the EASE score holds promise for further development. Prospective, multicenter studies are essential to validate its predictive performance and determine its added value compared to existing risk stratification tools. Integrating the score with advanced imaging techniques and electroanatomic mapping could further enhance its diagnostic precision. Additionally, embedding the EASE score into automated echocardiographic analysis software could streamline its implementation in clinical practice, facilitating broader adoption and more standardized patient assessment.

## 8. Conclusions

The EASE score provides a novel and integrative approach to pre-procedural evaluation in patients with paroxysmal atrial fibrillation undergoing catheter ablation. By combining six key echocardiographic parameters, covering atrial structure, function, conduction, and compliance, it delivers a comprehensive assessment of atrial health that extends beyond conventional volume-based metrics. The score’s strength lies in its clinical practicality: all components are derived from standard transthoracic echocardiography, making it easily implementable in routine practice without requiring advanced or invasive technologies.

This multidimensional model enhances risk stratification by identifying patients at high risk of post-ablation recurrence and those likely to benefit most from intervention. It also lays a foundation for more personalized treatment strategies, informed patient counseling, and potentially, improved long-term outcomes. In a field where variability in procedural success remains a challenge, the EASE score offers a standardized, reproducible tool to optimize therapeutic decision-making.

As a theoretical model, the EASE score invites further investigation and prospective clinical validation. Further validation in prospective, multicenter studies will be essential to confirm its predictive accuracy and to explore its integration with other diagnostic modalities such as MRI, biomarkers, and electroanatomic mapping. Nonetheless, the EASE score may represent a meaningful step forward in the pursuit of precision medicine in atrial fibrillation care.

## Figures and Tables

**Table 1:** EASE score risk categories and clinical interpretation. Stratifies patients into low, intermediate, or high risk of atrial fibrillation recurrence based on the extent of atrial remodeling.

EASE Score	Risk Category	Interpretation
0–3	Low	Minimal atrial remodeling; high likelihood of ablation success
4–8	Intermediate	Moderate remodeling; variable outcomes; consider enhanced monitoring
9–12	High	Extensive remodeling; increased risk of recurrence; consider adjunctive strategies

**Table 2:** Pathophysiological and electrophysiological correlates of EASE score parameters. Each metric reflects a distinct aspect of atrial remodeling with corresponding electrophysiologic features relevant to ablation outcomes.

EASE Parameter	Pathophysiological Role	Electrophysiological Correlate
LASr (Reservoir Strain)	Reflects LA compliance and fibrosis; lower values indicate impaired reservoir function	Corresponds to low-voltage zones (<0.5 mV), reduced CFAE density, and fibrotic substrate
PA-TDI (Atrial Conduction Time)	Indicates intra-atrial conduction delay and electromechanical dysfunction	Associated with prolonged activation time, interatrial dyssynchrony, and zig-zag conduction
LAVI (Volume Index)	Indicates chronic structural remodeling and LA dilation due to volume/pressure overload	Correlates with widespread CFAEs, multiple breakthrough sites, and complex activation patterns
E/e′ (Diastolic Pressure Estimate)	Reflects elevated LV filling pressure and LA pressure overload	Associated with prolonged electrogram duration, low voltage density, and post-ablation recurrence
Stiffness Index (E/e′/LASr)	Integrates diastolic burden and atrial compliance; higher values reflect stiffer LA	Overlaps with fragmented potentials, conduction heterogeneity, and low-voltage regions
LASct (Contractile Strain)	Reflects active LA contraction; low values indicate poor contractile function	Predictive of atrial standstill, late potentials loss, and reduced sinus rhythm maintenance post-ablation

**Table 3:** Baseline demographic and clinical characteristics of the study population. Continuous variables are presented as mean ± standard deviation (SD) or median with interquartile range (IQR).

Variable	Value
Age, years (mean ± SD)	64.3 ± 9.4
Sex, male (%)	75 (68.0%)
Hypertension (%)	68 (55.5%)
Diabetes mellitus (%)	22 (21.9%)
BMI, kg/m^2^ (mean ± SD)	27.2 ± 3.0
LA Volume Index, mL/m^2^ (mean ± SD)	34.2 ± 5.4
E/e′ ratio (mean ± SD)	9.0 ± 2.1
Follow-up duration, months (median (IQR))	14 [11–18]
EASE score—Low (0–3)	38 (29.7%)
EASE score—Intermediate (4–7)	61 (47.7%)
EASE score—High (8–12)	29 (22.6%)

## Data Availability

Data are available from the corresponding author on reasonable request.

## Abbreviations

The following abbreviations are used in this manuscript:

PAF	Paroxysmal Atrial Fibrillation
PVI	Pulmonary Vein Isolation
EASE	Echocardiographic Atrial Strain and conduction Evaluation (Score)
LAVI	Left Atrial Volume Index
LASr	Left Atrial Reservoir Strain
LASct	Left Atrial Contractile Strain
PA-TDI	Atrial Conduction Time Measured by Tissue Doppler Imaging
E/e′	Ratio of early mitral inflow to early diastolic mitral annular velocity
E/e′/LASr	Stiffness Index (a derived parameter combining diastolic function and strain)
EAT	Epicardial Adipose Tissue
